# Spatial–temporal vector abundance and malaria transmission dynamics in Nchelenge and Lake Mweru islands, a region with a high burden of malaria in northern Zambia

**DOI:** 10.1186/s12936-023-04746-5

**Published:** 2023-10-29

**Authors:** Mbanga Muleba, Keith J. Mbata, Jennifer C. Stevenson, Douglas E. Norris

**Affiliations:** 1 Tropical Diseases Research Centre, Ndola, Zambia; 2 Biological Sciences Department, School of Natural Sciences, University of Zambia, Lusaka, Zambia; 3 Macha Research Trust, Choma, Zambia; 4 The W. Harry Feinstone Department of Molecular Microbiology and Immunology, The Johns Hopkins Bloomberg School of Public Health, Baltimore, MD 21205 MD USA

**Keywords:** Malaria, *Anopheles funestus*, *Anopheles gambiae*, Nchelenge, Sporozoite rate, Entomological inoculation rate

## Abstract

**Background:**

Over a decade of vector control by indoor residual spraying (IRS) and long-lasting insecticidal nets (LLINs) distribution on the mainland, and only LLINs on islands had a minimal impact on disease burden in Nchelenge district, northern Zambia. *Anopheles funestus* and *Anopheles gambiae* are vectors known only from the mainland. Understanding vector bionomics in the district is necessary for planning and targeting effective vector control. This study aimed to provide information on abundance, seasonality, and *Plasmodium falciparum* sporozoite infectivity of malaria vectors in Nchelenge, including islands.

**Methods:**

Mosquitoes were collected in 192 CDC indoor light traps set in 56 households between January 2015 and January 2016. Morphological and molecular species identifications and *P. falciparum* circumsporoites by ELISA were performed. Mosquito counts and relative abundances from the islands and mainland were compared, and household factors associated with vector counts were determined.

**Results:**

A total of 5888 anophelines were collected during the study. Of these, 5,704 were female *Anopheles funestus *sensu lato (*s.l*.) and 248 female *An. gambiae s.l.* The highest proportion of *An. funestus* (n = 4090) was from Chisenga Island and *An. gambiae* (n = 174) was from Kilwa Island. The highest estimated counts per trap for *An. funestus s.l*. were from Chisenga island, (89.9, p < 0.001) and from the dry season (78.6, p < 001). For *An. gambiae* the highest counts per trap were from Kilwa island (3.1, p < 0.001) and the rainy season (2.5, p = 0.007). The highest estimated annual entomological inoculation rate was from Chisenga Island with 91.62 ib/p/y followed by Kilwa Island with 29.77 ib/p/yr, and then Mainland with 19.97 ib/p/yr.

**Conclusions:**

There was varied species abundance and malaria transmission risk across sites and seasons. The risk of malaria transmission was perennial and higher on the islands. The minimal impact of vector control efforts on the mainland was evident, but limited overall. Vector control intervention coverage with effective tools needs to be extended to the islands to effectively control malaria transmission in Nchelenge district.

## Background

The use of long-lasting insecticidal nets (LLINs), indoor residual spraying (IRS), artemisinin-based combination therapy (ACT), and intermittent preventive therapy in pregnancy (IPTp) in the last decade has led to successes against malaria in sub-Saharan Africa [1–5]. Sadly, not all areas receiving control interventions face an easy transition from malaria endemicity to malaria elimination [6]. For some high-transmission areas, the package of control interventions may need to be prescribed differently and specifically. In Zambia, Nchelenge District in the north is an area with high malaria transmission, with a sustained malaria prevalence between 30 and 50%, despite incremental coverage in control interventions comprised of ACTs, IRS, LLINs, IPTp since 2008, and more recently, integrated community case management (ICCM) [7, 8]. Studies from mainland Nchelenge have reported complex vector bionomics of two malaria vector species, *Anopheles funestus sensu stricto* (*s.s*.) and *An. gambiae s.s*. These studies have established that *An. funestus* is widely distributed and has a population peak during the dry season, while counts of *An. gambiae* remain relatively and consistently low throughout the year and are more abundant near Lake Mweru on the western border of the district [9–12]. The few genomics studies completed in Nchelenge have revealed the sympatric existence of Clades I and II of *An. funestus s.s*. and only S-form *An. gambiae*, with populations exhibiting varied insecticide susceptibility to carbamates, pyrethroids, and DDT [13–15]. It is unknown, however, to what extent these findings can be extended to the island settings within the district, where control measures are much more challenging to implement. The two big islands of the district, Kilwa Island and Chisenga Island, comprise nearly 13% of the district’s total population and, at the time of the study, had received only LLINs as malaria control interventions.

During the study period, cases of malaria reported between January and December 2015 for all age groups from Chisenga and Kilwa Islands rural health centres were 4, 282 and 8, 439, respectively (Nchelenge DHIS). These represented cases per 1000 people of 375 and 746 for Chisenga and Kilwa islands, respectively. Nchelenge Health Centre on the mainland reported 9360 cases with an incidence rate of 476/1000 (Nchelenge DHIS). For each health facility, almost half of the cases (2353 Chisenga Island, 4061 Kilwa Island, and 4477 Nchelenge) were in children 0–5 years old.

Given the enormity of malaria incidence in Nchelenge District, critical data is needed to better understand the underlying factors leading to the lack of success of malaria control in Nchelenge, especially in the context of malaria elimination [16]. Data on the two primary malaria vector species from the islands of Nchelenge is incredibly sparse. Published data was only from Kilwa Island mosquitoes, dating back to a 1926 report on non-malaria vectors [15]. The other known data on vectors came from a recent publication on *An. funestus s.s.* complete mitogenome [17], which included specimens collected from Kilwa Island during the initial preparatory stages of this study. This study aimed to expand ongoing malaria entomological studies in Nchelenge District to include the main islands in Lake Mweru. The focus was on vector seasonal and spatial abundance, *Plasmodium falciparum* sporozoite infection rates, and how these data may impact current and future malaria control strategies in the district. The study period was January 2015 to January 2016 as part of the Southern and Central Africa International Centres of Excellence for Malaria Research (ICEMR), a research collaboration set up to study malaria transmission and the impact of control interventions in Zambia, Zimbabwe, and the Democratic Republic of the Congo (DRC).

## Methods

### Study area

**Fig. 1:**
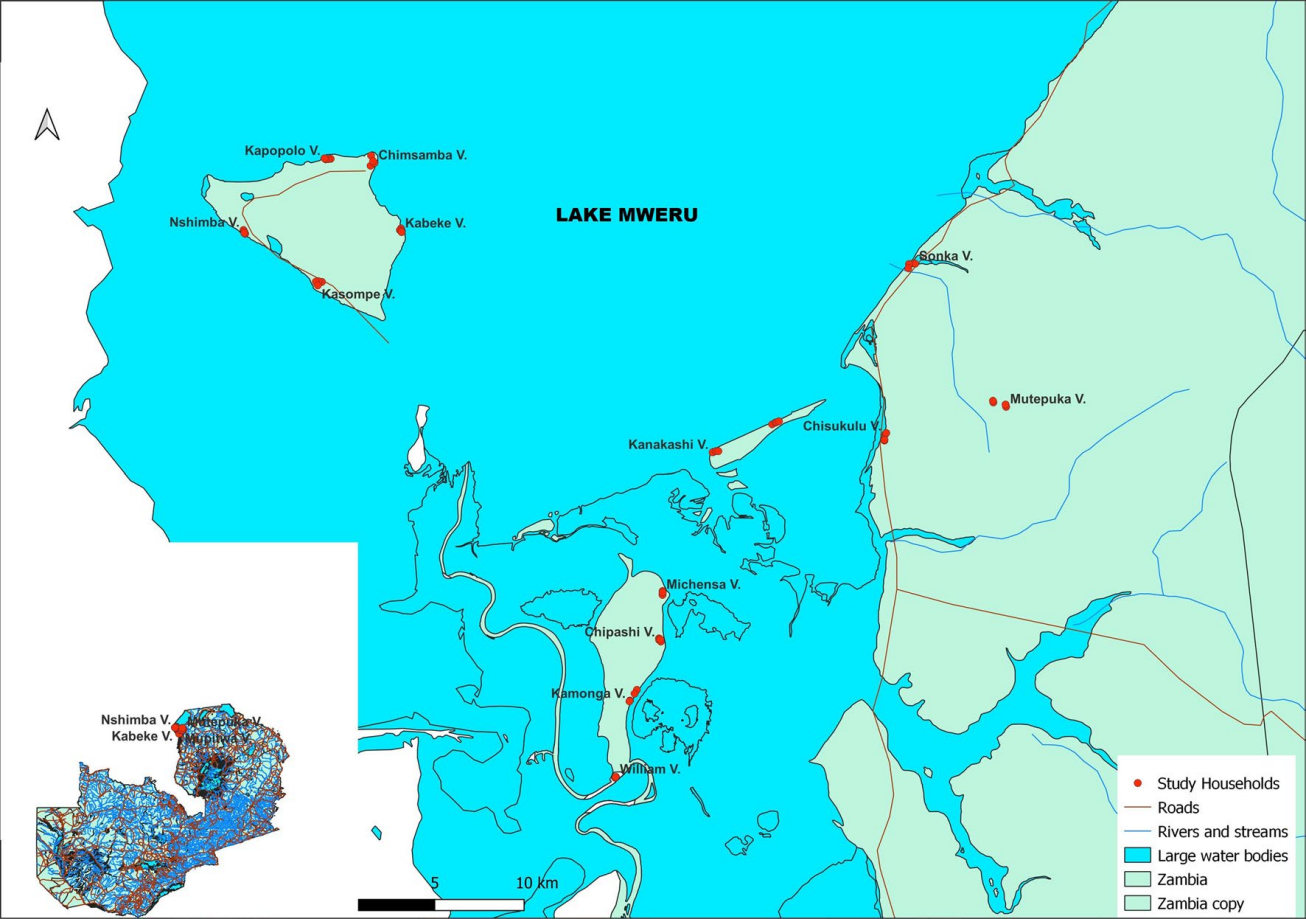
Map of Nchelenge and sampled households across the District (map composed using QGIS 3.22.7)

Nchelenge District is located in northern Zambia in Luapula Province (Fig. 1) at the mouth of the Luapula River, forming part of the Luapula-Mweru delta. Lake Mweru and the Luapula River form the border with the DRC, and the Luapula-Mweru system interface includes vast swamps and lagoons and a number of islands. Prominent among the islands on the Zambian side of Lake Mweru are Chisenga, Kilwa, and Isokwe (Fig. 1). The mainland along Lake Mweru hosts a network of shallow streams with slow-moving waters and expansive vegetation cover. Mosquitoes are hypothesised to breed along the edges of these streams, which remain inundated for extended periods of the year.

Nchelenge is largely a rural district with an estimated human population at the time of the survey of 147,927 [18]. The mainland constituted about 77% of the total district population. The island populations by head count from the district health office were estimated at 11409 for Chisenga Island, 3501 for Isokwe Island, and 11305 for Kilwa Island (Nchelenge DHO). The majority of the island's inhabitants are involved in fishing, while also engaging in subsistence farming. The formal housing structures with metal roofing are typical of the district's urban areas. The rural areas comprise houses with mud walls and thatch roofing. Makeshift houses, which expose the inhabitants to mosquito bites, are characteristic of farming fields on the mainland and nearly all houses on Isokwe Island and in fishing camps on the islands. Seasonal human movement between the interior mainland, lakeside, and islands is common. On the mainland, the beginning of the rainy season necessitates the shift to the interior, where there are farming fields. The rainy season is usually between November and May, and the dry season comes between June and September. The collections conducted during the rainy season in 2015 were defined as rainy season 1, and those done in 2016 were designated rainy season 2.

### Vector control

Indoor residual spraying and bed net distribution form the main vector control interventions conducted in Nchelenge by the national programme. The IRS programme is normally conducted between September and December of each year. During the study period, IRS with pirimiphos-methyl (Actellic® 300CS) was conducted in 2014 and 2015, and a mass campaign for LLIN (Olyset®) distribution took place in October 2014.

### Selection of study households

Satellite imagery of the three islands from Google Maps and an ARCGIS image of mainland Nchelenge District were overlaid with 1 km^2^ grids [19]. Grids with human habitation were enumerated, and study households were selected by simple random sampling. On the ground, houses within a selected grid (hereafter referred to as a village) were enumerated, and four houses were randomly selected. A total of 56 houses across 14 villages in the study area were subsequently selected and their geographic coordinates recorded (using a Garmin OREGON® 450 GPS gadget). The 14 villages were distributed as follows: Chisenga Island, 4; Isokwe Island, 2; Kilwa Island, 5; and Mainland, 3.

### Mosquito collection

The collection of mosquitoes was conducted using CDC light traps hung indoors approximately 150 cm above the floor at the foot end of the sleeping space. A total of 192 traps were set indoors during the entire study period. A total of five rounds of collection were conducted, as follows: First rainy season: 32 households (HH) in January 2015 and 36 HH in March 2015. During the dry season, 37 HH were recorded in July 2015 and 42 HH in October 2015. Finally, the second rainy season 44 HH in January 2016 Each of the selected households was sampled a minimum of one and a maximum of five times (Table 1) over the entire sampling period. Only data from 42 households, a total of 165 trap nights (marked with § in Table 1) sampled 3–5 times, were included in the analysis of malaria vectors’ spatial and temporal abundances.

**Table 1:** Distribution of households and sampling efforts across sites over the course of the study

No. of times sampled	No. of households from study site				
	Chisenga island	Isokwe island	Kilwa island	Mainland	Total sampling
5	4^§^	0	3^§^	5^§^	60
4	7^§^	0	5^§^	3^§^	60
3	4^§^	0	7^§^	4^§^	45
2	1	6	4	0	22
1	0	2	1	0	3
Total households	16	8	20	12	

^§^Number of households included in analysis of seasonal and spatial abundance

### Processing of collected mosquitoes

All anopheline mosquitoes collected were morphologically identified by standard identification keys to species groups upon collection in the field [20, 21]. These were individually packed into 0.5-mL centrifuge tubes with desiccant for further laboratory processing. A representative sub-sample of 20% of specimens morphologically identified as female *An. funestus sensu lato* (*s.l*.) and *An. gambiae s.l.* were processed for polymerase chain reaction (PCR) and circumsporozoite ELISA.

Mosquitoes were dissected at the thoracic and abdominal joints into the head, thorax, and abdomen. DNA was extracted from either a leg or abdomen and processed by PCR for species identification [22, 23]. The M and S molecular form determinations of *An. gambiae s.s.* were completed by PCR, as previously described [24].

TaqMan PCR, as previously described [25], identified the *An. funestus* clades. The *P. falciparum* sporozoite infections were determined by the circumsporozoite ELISA assay pf-210 [26, 27]. To remove false positives, the homogenates of all the assays that tested positive on the first run of circumsporozoite ELISA were boiled at 100 °C for ten minutes [28], and the ELISA was repeated. The *P. falciparum* sporozoite rate was calculated as the number of mosquitoes detected with *P. falciparum* sporozoites out of the total tested by circumsporozoite ELISA [29]. The biting rate was taken as previously done by Das et al. [10], taking into account the total number of people in the households. Annual entomological inoculation rates were estimated as the product of the biting rate and sporozoite rate multiplied by 365 days.

### Data analysis

The mosquito counts were entered in Excel sheets and exported to R and STATA 15 (Release 15, College Station, TX: Stata Corp LLC) for analysis. Morphological data were used for the analyses of species abundance and distribution. Only data from households sampled at least three times were included in the analyses of the seasonal and spatial abundance of vector species. The species PCR data were used to compare vector species infection rates and their spatial and seasonal occurrence.

Variations in total female anophelines collected from each site were evaluated separately for each of the two vector species by multilevel mixed-effects negative binomial regression with household as a random covariate. The covariates of fixed effects were season and site of collection. Marginal effects and means of vector counts were predicted for the season and site of collection. The *P. falciparum* sporozoite infection rates in *Anopheles* species were seasonally and spatially compared and predicted by logistic regression analysis. QGIS version 3.10.2 was used to create maps and plot proportions of mosquito counts as well as *P. falciparum* infection rates.

## Results

### Proportions of major vectors in the study area

**Fig. 2:**
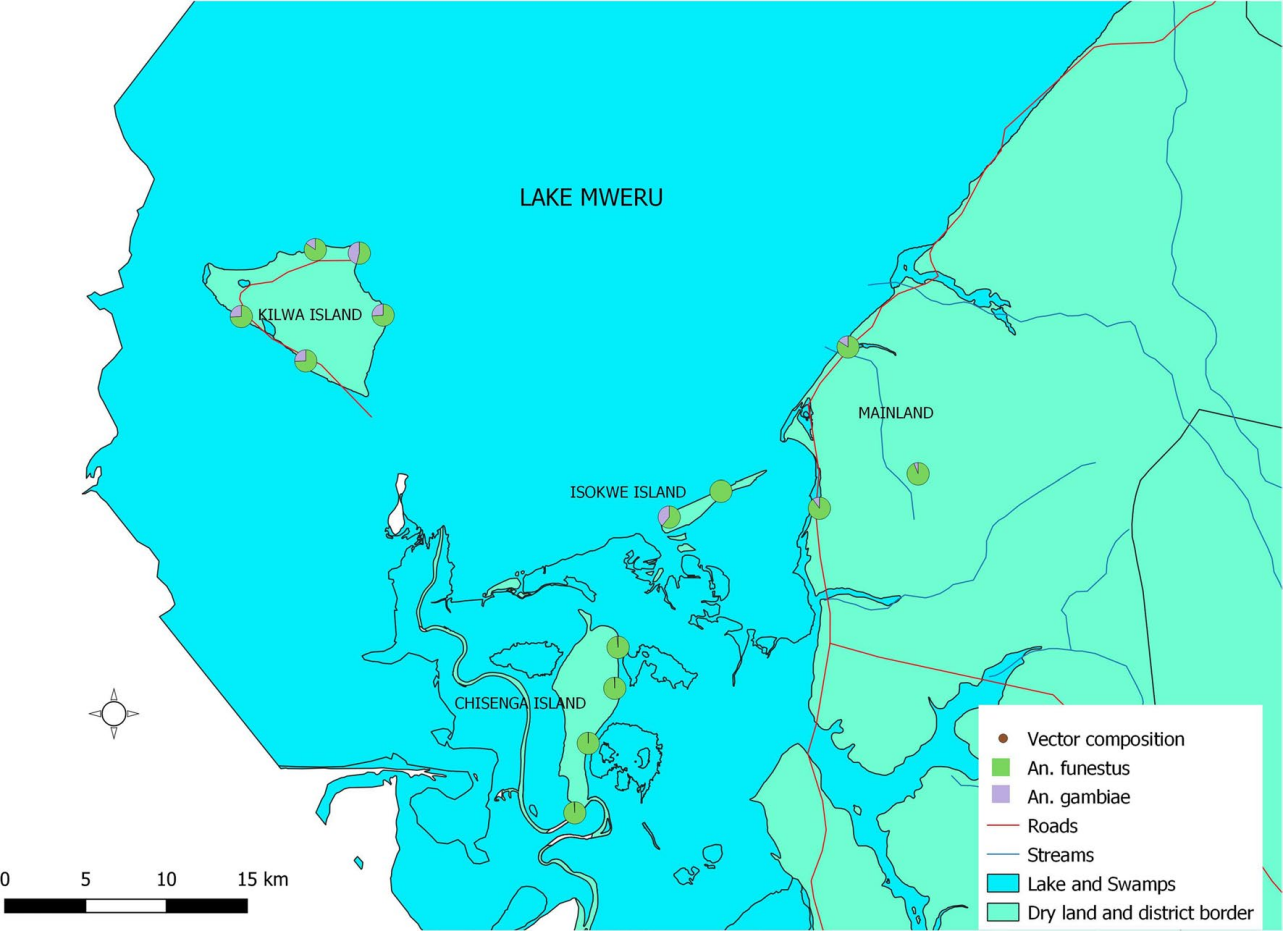
Mean composition of the primary malaria vectors *An. funestus s.l.* and *An. gambiae s.l.* across the study area (map composed using QGIS3.22.7)

A total of 5888 Anopheles mosquitoes were collected indoors from 56 households in 14 villages (Fig.2). A total of 858 people slept in the houses on the nights of the mosquito sample collection. Of the total collection, 5579 were female anophelines, distributed as follows: *Anopheles funestus s.l.* = 5323, *An. gambiae s.l.* = 249, *Anopheles coustani s.l.* = 4, and unidentified = 3.

The count data of the two major malaria vectors were highly skewed, with 31 traps recording zero *An. funestus s.l.* collections and 97 traps recording zero *An. gambiae s.l.* collections. The maximum count of *An. funestus s.l.* in a single light trap was 497 from Chisenga Island, and the maximum for *An. gambiae s.l.* was 30 from Kilwa Island (Table 2).

**Table 2:** Proportions of the total vectors collected and the maximum count in a single light trap in different sites

Site	Variable	*An. funestus s.l*	*An. gambiae s.l*
Chisenga island	Trap nights	60	60
	Total mosquitoes collected	4090	26
	Maximum	497	9
Kilwa island	Trap nights	56	56
	Total mosquitoes collected	552	174
	Maximum	88	30
Mainland	Trap nights	49	49
	Total mosquitoes collected	598	38
	Maximum	242	13

*Anopheles funestus s.l.* count (n = 5240) were dominant over *An. gambiae s.l.* (n = 238) across all study sites (Table 2). Chisenga Island had the highest proportion of *An. funestus s.l.* (n = 4090), and Kilwa Island had the highest proportion of *An. gambiae s.l.* (n = 174).

### Factors associated with malaria vector counts

The estimated counts per trap for the two vector species across the study sites ranged from 8.602 to 89.97 for *An. funestus s.l.* and 0.499 to 3.083 for *An. gambiae s.l.* (Table 3). On the other hand, seasonal counts per trap for these vectors ranged from 4.869 to 78.606 for *An. funestus* and 0.818 to 2.548 for *An. gambiae* (Table 3).

**Table 3:** Factors associated with the malaria vector counts in CDC light traps set indoors

Covariate	*An. funestus* s*.l*			*An. gambiae s.l*		
	IRR/Predicted mean count	95% CI	p-value	IRR/Predicted mean count	95%CI	P-value
Season	Ref.					
Dry						
Rainy 1	0.189	0.115–0.311	< 0.001*	0.77	0.365–1.627	0.494
Rainy 2	0.069	0.038–0.124	< 0.001*	1.868	0.858–4.065	0.404
Site						
Mainland	Ref.					
Chisenga Island	9.341	3.516–24.820	< 0.001*	0.805	0.282–2.300	0.685
Kilwa Island	0.877	0.329–2.335	0.793	4.573	1. 619–12.915	0.004*
Spatial abundance						
Chisenga Island	89.944	46.995–132.893	< 0.001*	0.499	0.175–0.823	0.003*
Kilwa Island	9.461	4.871–14.051	< 0.001*	3.083	1.546–4.620	< 0.001*
Mainland	8.602	0.578–16.626	0.036*	0.639	0.061–1.217	0.030*
Seasonal abundance						
Dry	78.606	38.997–118.215	< 0.001*	1.382	0.510–2.254	0.002*
Rainy 1	16.613	8.244–24.983	< 0.001*	0.818	0.378–1.258	< 0.001*
Rainy 2	4.869	1.815–7.923	0.002*	2.548	0.686–4.410	0.007*

*IRR*  incidence rate ratio

*Shows results were significant at 95% confidence level

The *An. funestus* counts were strongly associated with Chisenga Island (IRR = 9.341, p < 0.001), and those of *An. gambiae* were so with Kilwa Island (IRR = 4.573, p = 0.004). There was a significant reduction in the estimated *An. funestus* count during rainy seasons 1 and 2, IRR = 0.189, p < 0.001, and IRR = 0.069, p < 0.001, respectively (Table 3). The *An. gambiae* count reduced during rainy season 1 (IRR = 0.770, p = 0.494), but the count increased during rainy season 2 (IRR = 1.868, p = 0.115) (Table 3).

### Molecular identifications

Molecular species identifications by PCR on a sub-sample (1108 females) successfully amplified 978 (88%) of the specimens processed. DNA from 131 specimens did not amplify.

Identification of species by PCR showed *An. funestus s.s*. (n = 773) and *Anopheles leesoni* (n = 10) as members of the *An. funestus* group. Further mitochondrial DNA analysis of *An. funestus s.s.* revealed the existence of Clade I (n = 736) and Clade II (n = 37), as previously reported [13]. The *An. gambiae s.l.* PCR analysis of specimens revealed only *An. gambiae s.s*. (n = 195). Further processing by TaqMan hydrolysis PCR to determine the M-form (*Anopheles coluzzii*) and S-forms revealed the S-form of *An. gambiae s.s.* as the only member of the complex collected.

### *Plasmodium falciparum* infection rates in the vectors

A total of 30 specimens gave a positive *P. falciparum* sporozoite ELISA result on the first run. When the homogenates were heated to remove false positives, 25 still came out positive. The *P. falciparum* circumsporozoite ELISA showed positive results in *An. funestus s.s.* Clade I, 18 (n = 736), and *An. gambiae,* 7 (n = 195). No sporozoites were detected in *An. funestus s.s.* Clade II (n = 37) or *An. leesoni* (n = 10).

In the study sites, *P. falciparum* infections were found in both primary vectors, *An. funestus s.s.* and *An. gambiae s.s*., from Kilwa Island and the mainland. For Isokwe Island and Chisenga Island, only *An. funestus s.s.* was found infected with *P. falciparum*. Using the foraging rates based on the count of mosquitoes and household occupants the annual entomological inoculation rates (EIR) were estimated for each collection site. The EIR for Chisenga Island was 91.62 infectious bites per person per year (ib/p/yr), all of which came from *An. funestus s.s.* Kilwa Island's EIR was 29.77 ib/p/yr, with species contributions of 21.21 ib/p/yr and 8.57 ib/p/yr from *An. funestus s.s*. and *An. gambiae s.s*., respectively. Mainland EIR was estimated to be 19.97 ib/p/yr, with *An. funestus s.s*. contributing 18.12 ib/p/yr and *An. gambiae s.s*. giving 1.85 ib/p/yr.

**Fig. 3:**
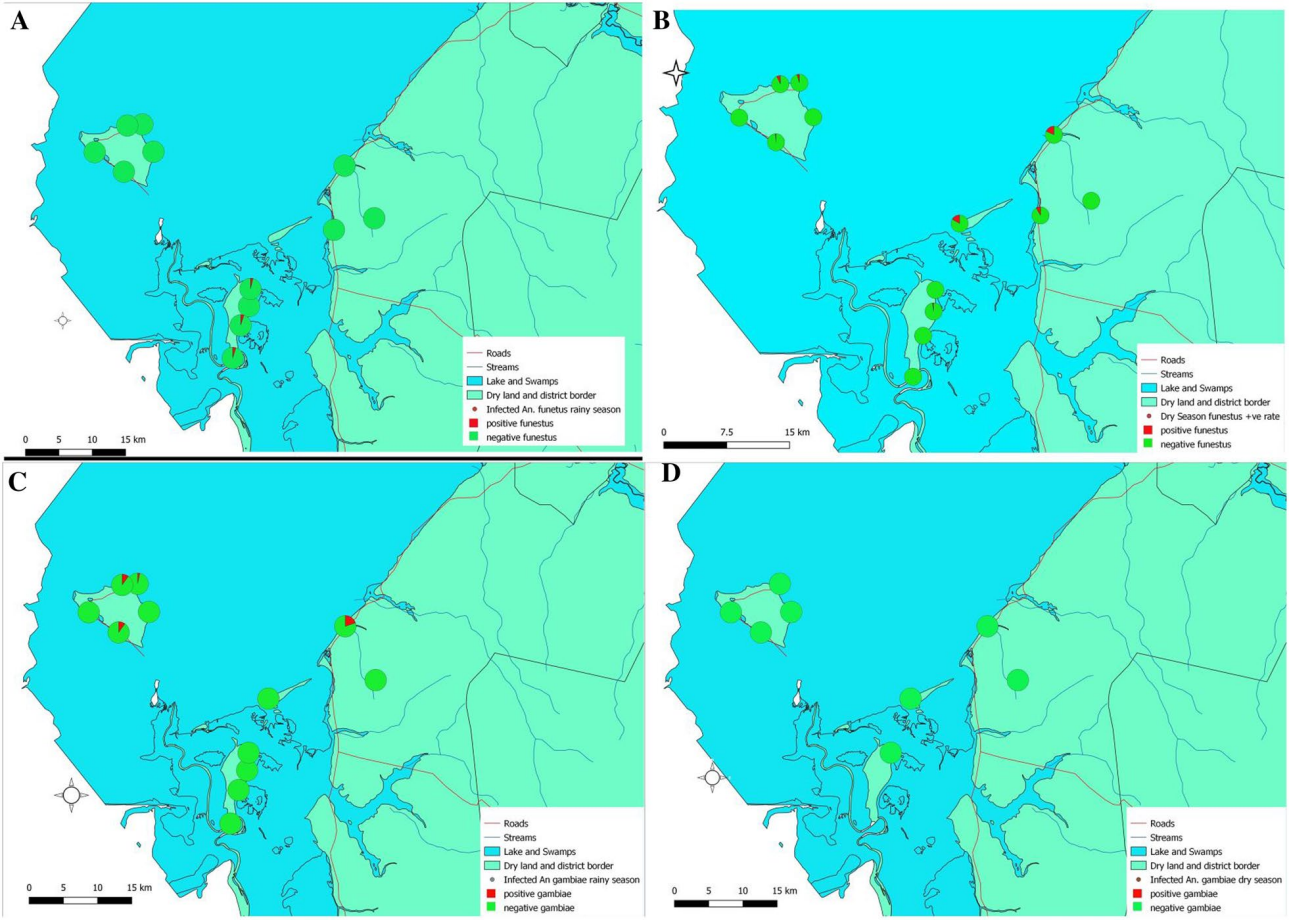
Spatial and seasonal distribution of *P. falciparum* sporozoite infections in the primary vectors across study sites. **A** and **B** represent infections in *An. funestus s.l.* in the rainy and dry seasons, respectively. **C** and **D** represent infections in *An. gambiae s.l.* rainy and dry seasons, respectively (maps composed using QGIS 3.22.7)

Seasonally, *P. falciparum* sporozoites-infected vectors were detected in both dry and rainy seasons (Fig. 3). In the rainy season, *P. falciparum* sporozoites were detected among *An. funestus s.s.* samples from Chisenga Island (Fig. 3A) and in *An. gambiae s.s*. samples from Kilwa Island and the mainland (Fig. 3C). In the dry season, *P. falciparum* sporozoite infections were detected in *An. funestus* s.s. from Isokwe Island, Kilwa Island, and the mainland (Fig. 3B). No dry-season *P. falciparum* sporozoites were detected in *An. gambiae s.s.* from any site (Figure 3D).

Seasonal and spatial comparisons in *P. falciparum* infection rates by logistic regression (Table 4) in the two vector species revealed 54% lower odds of infection in *An. gambiae s.s.* compared to *An. funestus s.s.* Clade I (OR = 0.460, 95% CI: 0.184–1.150, p = 0.097). The odds of infection with *P. falciparum* in vectors were lower on Chisenga Island compared to the mainland (OR = 0.646, 95% CI: 0.198–2.109, p = 0.649). The odds of infection in vectors were higher on Isokwe Island compared to the mainland (OR = 3.564, 95% CI: 0.506–25.100, p = 0.202), and higher on Kilwa Island compared to the mainland (OR = 1.322, 95% CI: 0.388–4.506, p = 0.655).

**Table 4:** *Plasmodium falciparum* infection analysis by logistic regression

Covariate	OR	95% CI	p-value	Predicted SR	95% CI	p-value
Vector						
* An. funestus s.s*	Ref.			0.042	0.016–0.068	0.001*
* An. gambiae s.s*	0.46	0.184–1.50	0.097	0.02	0.004–0.036	0.014*
Season						
Dry	Ref.			0.021	0.008–0.035	0.002*
Rainy 1	0.722	0.216–2.413	0.596	0.015	0.000–0.034	0.092
Rainy 2	3.596	1.593–8.118	0.002*	0.073	0.026–0.119	0.002*
Site						
Mainland	Ref.			0.022	0.000–0.045	0.056
Chisenga Island	0.646	0.200–2.109	0.469	0.014	0.003–0.026	0.017*
Isokwe Island	3.564	0.506–25.100	0.202	0.075	0.000–0.215	0.173
Kilwa Island	1.322	0.388–4.506	0.655	0.029	0.010–0.048	0.003*

*OR* odds ratio, *SR*  sporozoite rate

*Shows results were significant at 95% confidence level

The estimated probability of *An. funestus s.s.* Clade I infection with *P. falciparum* sporozoites during the study was 0.042 (95% CI: 0.16-0.68, p = 0.001). The estimated probability of infection in *An. gambiae s.s.* was 0.020 (95% CI: 0.004–0.036, p = 0.014). Seasonal predicted probabilities of infection with *P. falciparum* in vectors were: dry season 0.021 (95% CI: 0.008–0.035, p = 0.002), rainy season 1, 0.015 (95% CI: 0.000–0.034, p = 0.092), and rainy season 2, 0.073 (95% CI: 0.026–0.119, p = 0.002).

## Discussion

This study determined the spatial and seasonal abundances of the two primary vectors of malaria in Nchelenge, including islands. The infection with *P. falciparum* sporozoites in the two primary vectors of malaria, *An. funestus s.s.* and *An. gambiae s.s.,* in Nchelenge was determined. There were significant differences in the abundance of *An. funestus s.l.* on Chisenga Island and of *An. gambiae s.l.* on Kilwa Island compared to the mainland. The *An. funestus s.l.* was more abundant during the dry season, and the *An. gambiae* was abundant during the rainy season. Predicted probabilities for *P. falciparum* infection were significant for both *An. gambiae s.s*. and *An. funestus s.s.*, with *An. funestus s.s*. showing a slight edge over *An. gambiae s.s*. When the two vectors were considered together, the predicted probability of a malaria vector for infection with *P. falciparum* during both the dry and rainy seasons was significant. The estimated annual entomological inoculation rate, which is a measure of human exposure to the risk of malaria transmission, was highest on Chisenga Island with 91.62 ib/p/yr, followed by Kilwa Island with 29.77 ib/p/y, and lastly Mainland with 19.97 ib/p/yr.

These results show a dynamic entomological picture driving malaria transmission in the district. The results obtained here both confirmed what was already known about Nchelenge and provided new insights about the malaria vectors on the islands. At the time of this study, the only form of vector control for the islands was net distribution. *Anopheles funestus s.l.* dominated and was more abundant during the dry season, in agreement with previous findings [10, 12]. This study went further to show that Chisenga Island had disproportionally higher densities of *An. funestus s.l.* compared to either Kilwa Island or the mainland. The highest counts of *An. gambiae s.l.* were recorded from Kilwa Island. These findings seem to reflect local differences in the ecologies of these different study sites, which may be important for targeting control interventions [30, 31].

The findings of this study are in support of prior studies that assessed household risk factors for mosquito abundance on the mainland [9, 12]. Mosquito counts found in this study suggest that high bed net usage could be driven by high indoor densities of pyrethroid-resistant mosquitoes [9]. The findings from this study indicate that this scenario would be widespread across Nchelenge, including islands. These scenarios would raise concern as to the efficacy of the use of LLINs as the only vector control intervention for the islands in Nchelenge District. Other interventions, including the use of PBO-synergized LLINs designed to address the high rates of pyrethroid resistance, need to be included. Other household factors, such as thatch roofing and open eaves indicative of poor construction, allowed easier mosquito entry. Improved household structures should be considered a compliment to or integrated into the vector control programme for the district. Studies done in Cameroon, The Gambia, and Kenya have demonstrated that simple and low-cost improvements to house structure would be effective at preventing mosquito entry and reducing indoor densities [32–35].

The parasite sporozoite rates observed suggested variations in spatial intensity but a sustained, perennial risk of vectors infected with *P. falciparum* sporozoites. *Anopheles funestus s.s.* contributed to transmission at all study sites during the dry season and on Chisenga Island during the rainy season. In contrast, *An. gambiae* appeared to drive transmissions during the rainy season on Kilwa Island and the mainland. The mainland and Kilwa Island were the only sites where both vector species were found infected with *P. falciparum*. Despite their relatively low numbers, *An. gambiae* had comparable rates of *P. falciparum* sporozoite infections with the more abundant *An. funestus s.s.* on both Kilwa Island and the mainland. This demonstrated that *An. gambiae* made substantial contribution to malaria transmission in Nchelenge, a fact that needs to be considered when designing and implementing vector control interventions. This study found no *P. falciparum*-infected Clade II *An. funestus s.s.* This was in contrast with the prior study by Choi *et al.* [13] on the mainland that could not attribute any difference in malaria transmission to either Clade, and thus our findings could be attributed to sample size.

Two previous studies, one intensive and another extensive, both conducted in 2012 and 2013, made comparable estimates of the EIRs for the Nchelenge mainland with different calculations of the human biting rate [10, 12]. The EIR estimated for the mainland in the current study was much lower for both *An. funestus s.s.* and *An. gambiae s.s.* The EIR is an important indicator of the intensity of malaria transmission in an area. A reduction in EIR is one of the indices used to measure the impact of vector control activity [36, 37]. This study followed a mass LLIN distribution campaign and two years of IRS with pirimiphos-methyl on the mainland. Two studies on the mainland that looked at the effects of three years of IRS with pirimiphos-methyl on parasite prevalence and the number of malaria vectors found that the number of vectors in sprayed homes and targeted areas went down, but parasite prevalence did not change much [38, 39]. The present study findings on the mainland suggest reduced EIR estimates, which seem to agree with the reduced vector abundances and parasite prevalence reported during the study period.

Twelve months of concomitant malaria incidence data were obtained from health centres (HCs) servicing the three individual collection study sites. According to these data, malaria incidence was highest on Kilwa Island HC, then on the mainland Nchelenge HC, and lowest on Chisenga Island HC. In contrast, Chisenga Island had the highest estimated EIR, followed by Kilwa Island, and then the mainland. In an area with high vector counts, the discrepancy between estimated EIR and reported malaria cases may be due to the unreported cases that get treated at home within communities. No prevalence studies were conducted in the present study, nor had they been conducted before on the islands. For the islands in Nchelenge, local factors including ecology, demographics, malaria control use, and mosquito bionomics would need to be considered in the estimation and interpretation of the EIR. Monitoring how EIRs would be affected would be important in the evaluation of the scale-up of the malaria control package on the islands of Nchelenge.

## Conclusions

Consistent with prior studies in Nchelenge District, this study reports a high abundance of the primary vector, *An. funestus s.s.,* and a relatively low abundance of *An. gambiae*, including island sites for the first time. In sites and periods where both vector species were involved in malaria transmission, sporozoite infection rates were comparable, thereby adding to a higher risk for malaria transmission. The combination of IRS and LLINs on the mainland might have a very minimal impact on malaria transmission. Sustained vector control at all the sites should be emphasised with expanded coverage of intervention on the islands. Non-chemical mosquito bite control and prevention options should be considered for Nchelenge. Continued research on the vectors, the parasites, and the human host at different sites is required to fully understand the burden and effectively target control in different areas of Nchelenge.

## Data Availability

Raw data on the mosquito collections and entomological indices is available upon reasonable request from the corresponding author.

## Abbreviations

ACT: Artemisinin-based combination therapy
ARCGIS: Aeronautical Reconnaissance Coverage Geographic Information System
CDC: Centres for Disease Control and prevention
DDT: Dichloro-diphenyl-trichloroethane
DHIS: District Health Information System
DHO: District Health Office
DNA: Deoxyribonucleic Acid
DRC: Democratic Republic of Congo
EIR: Entomological Inoculation Rate
ELISA: Enzyme-linked Immunosorbent Assay
ERC: Ethics Review Committee
GPS: Global Position System
HC: Health Centre
HH: Household
ICCM: Integrated Community Case Management
ICEMR: International Centres of Excellence for Malaria Research
IPTp: Intermittent Preventive Therapy in Pregnancy
IRS: Indoor Residual Spraying
ITNs: Insecticide-Treated Nets
LLINs: Long-lasting Insecticidal Nets
M & S: Mopti & Savanna strain
Mw: Williams Mean
OR: Odds ratio
PCR: Polymerase chain reaction
QGIS: Quantum Geographic Information System
STC: Scientific and Technical Committee
TDRC: Tropical Diseases Research Centre
